# Effect of Composition on Polarization Hysteresis and Energy Storage Ability of P(VDF-TrFE-CFE) Relaxor Terpolymers

**DOI:** 10.3390/polym13081343

**Published:** 2021-04-20

**Authors:** Yusra Hambal, Vladimir V. Shvartsman, Daniil Lewin, Chieng Huo Huat, Xin Chen, Ivo Michiels, Qiming Zhang, Doru C. Lupascu

**Affiliations:** 1 Institute for Materials Science and Center for Nanointegration Duisburg-Essen (CENIDE), University of Duisburg-Essen, 45141 Essen, Germany; yusra.hambal@uni-due.de (Y.H.); daniil.lewin@uni-due.de (D.L.); huo.chieng@stud.uni-due.de (C.H.H.); ivo.michiels@uni-due.de (I.M.); doru.lupascu@uni-due.de (D.C.L.); 2 Department of Electrical Engineering, Materials Research Institute, The Pennsylvania State University, University Park, State College, PA 16802, USA; xzc29@psu.edu (X.C.); qxz1@psu.edu (Q.Z.); 3 Department of Materials Science and Engineering, Materials Research Institute, The Pennsylvania State University, University Park, State College, PA 16802, USA

**Keywords:** polymers, relaxors, energy storage, P(VDF-TrFE-CFE)

## Abstract

The temperature dependence of the dielectric permittivity and polarization hysteresis loops of P(VDF-TrFE-CFE) polymer films with different compositions are studied. Among them, the three compositions, 51.3/48.7/6.2, 59.8/40.2/7.3, and 70/30/8.1, are characterized for the first time. Relaxor behavior is confirmed for all studied samples. Increasing the CFE content results in lowering the freezing temperature and stabilizes the ergodic relaxor state. The observed double hysteresis loops are related to the field-induced transition to a ferroelectric state. The critical field corresponding to this transition varies with the composition and temperature; it becomes larger for temperatures far from the freezing temperature. The energy storage performance is evaluated from the analysis of unipolar polarization hysteresis loops. P(VDF-TrFE-CFE) 59.8/40.2/7.3 shows the largest energy density of about 5 J·cm^−3^ (at the field of 200 MV·m^−1^) and a charge–discharge efficiency of 63%, which iscomparable with the best literature data for the neat terpolymers.

## 1. Introduction

**Figure 1:**
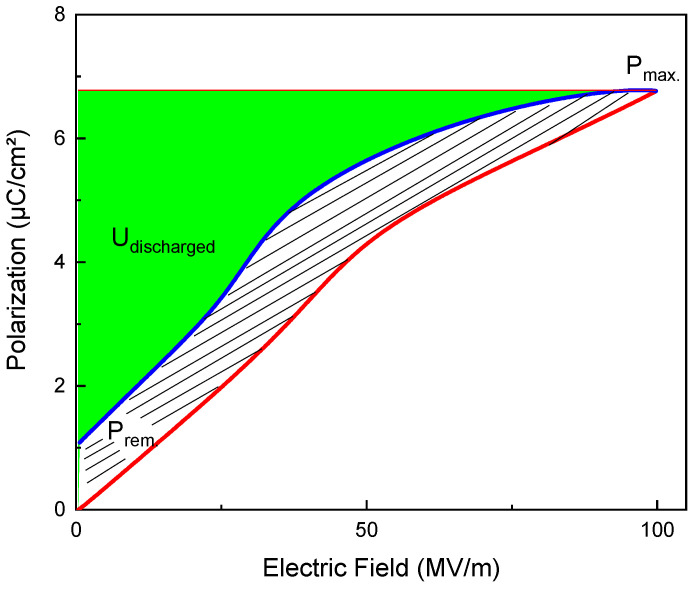
A unipolar polarization hysteresis loop. The green area corresponds to the recoverable energy density. The hatched area yields losses in the storage process.

Alternative technologies in the energy generation sector, miniaturization in the electronics industry, and electric mobility have opened up many doors for advancements in the field of energy storage [1]. Due to the high dielectric strength, various dielectric polymers, such as polypropylene, polycarbonate, and polyethylene terephthalate, were commercially used for decades in thin film and thick film capacitors, all showing a linear dielectric response and thus a plain capacitive behavior [2,3,4]. For a non-linear or hysteretic material, the stored energy density in a polarization–electric field graph is given by the area between the *charging branch* of a dielectric displacement—electric field hysteresis loop and the dielectric displacement axis (Figure 1).

(1) $$U_{stored}=\int_{0}^{D_{max}}\vec{E}\;\left(\vec{D}\right)\cdot d\vec{D},$$

with the dielectric displacement given by the polarization $\vec{P}$ of the material and the dielectric permittivity of free space $\vec{D}=\varepsilon_{0}\cdot \vec{E}+\vec{P}\left(\vec{E}\right)$.

Therefore, polymers exhibiting high polarizability such as ferroelectric polyvinylidene fluoride P(VDF) and its copolymers with trifluoroethylene (TrFE), hexafluoropropylene (HFP), chlorofluoroethylene (CFE), and chlorotrifluoroethylene (CTFE), as well as blends between them or with other linear dielectric polymers, were studied for energy storage applications [5,6,7,8]. Ideally, all this stored energy should berecoverable, which entails that a remanent contribution to polarization should be small. In ferroelectric polymers, saturation and remanent polarization often do not differ much, which is unfavorable for the recoverable energy storage density which is defined by the area between the discharging part of the D–E hysteresis loop and the dielectric displacement axis.

(2) $$U_{discharged}=\int_{D_{rem}}^{D_{max}}\vec{E}\;\left(\vec{D}\right)\cdot d\vec{D}$$

Therefore, materials with a high polarizability and a slim hysteresis loop are more suited for energy storage applications. Such a combination of properties is typical for relaxor ferroelectrics or shortly speaking relaxors [9].

In relaxors, the transition into a long-range ordered ferroelectric state is hindered by structural or charge disorder.Therefore, polarization is correlated at the local scale within polar nanoregions (PNRs) having asize of a few nanometers [9]. At high temperatures, the dipole moments of these PNRs are dynamic and are easily rotated by an electric field, facilitatinga large polarizability of relaxors. When the field is removed, the PNRs return to a disordered state, resulting in a small remanent polarization. To distinguish from the paraelectric state, this state is also called the ergodic relaxor state.

In order to achieve relaxor behavior in P(VDF-TrFE) co-polymers, various techniques, such as electron beam and γ-beam irradiation, mechanical stretching, and defect modification were implemented. By defect modification, another bulky monomer such as chlorofluoroethylene (CFE), hexafluoropropylene (HFP), or chlorotrifluoroethylene (CTFE) is incorporated into P(VDF-TrFE) [10,11,12,13]. It was reported that terpolymers P(VDF-TrFE-CFE) with the VDF/TrFE molar ratio below 75/25 and the molar amount of CFE > 4 mol% exhibit relaxor behavior with a broad and frequency-dependent peakof the dielectric permittivity [10,11,14]. For P(VDF-TrFE-CFE), both single hysteresis loops (SHL) [15,16], which are typical for relaxors, and double hysteresis loops (DHL) [12] were reported. The DHL behavior was attributed to a field-induced phase transition. The double hysteresis behavior of the polymer relaxors is not well-understood yet [17]. It was shown that both SHL and DHL behavior can be observed in the same polymer composition depending on the synthesis conditions [13].

Small remanent polarization and slim hysteresis make P(VDF_x_-TrFE_1-x_-CFE_y_) terpolymers attractive for energy storage applications [18,19]. A large electrocaloric effect was also recently reported in these materials [20,21]. However, among the many possible compositions, only a few have been studied so far. An investigation of dielectric and ferroelectric behavior in a broader concentration range should help to understand the mechanism of the formation of the relaxor state and the relationship between the property and composition in these polymers.

In the present work, we have studied temperature dependence of dielectric permittivity and polarization of five different P(VDF_x_-TrFE_1-x_-CFE_y_) compositions (Figure S1). To our knowledge, for the three compositions: 51.3/48.7/6.2, 59.8/40.2/7.3, and 70/30/8.1, such studies are reported for the first time. The relaxor behavior of the terpolymers is analyzed. The effect of the CFE content on the relaxor behaviour, the shape of the hysteresis loops, and the energy storage capability of the studied compositions is discussed.

## 2. Materials and Methods

The terpolymer powders used in this study, which were synthesized via suspension polymerization [13], were purchased from Piezotech, Pierre-Bénite, France. The studied compositions are listed in Table 1.

The polymer films were prepared by the drop-casting method. The polymer powder was dissolved in Dimethylformamide (DMF; VWR Chemicals, Radnor, PA, USA) and stirred overnight at room temperature. The concentration of the solution was set to 20 g L^−1^. The polymer solution was filtered and drop-coated onto a glass substrate (Corning Inc. Corning, New York, NY, USA). The coating was dried at 60 °C for 20 h, followed by annealing under vacuum (260 mbar) at 100 °C for 8 h and slowly cooled down to room temperature. The film with the substrate was then immersed in distilled water for some time, peeled off, and dried using a fiberless tissue. The final thickness of the freestanding films was around 20 µm.

To perform electric measurements, silver electrodes were deposited onto the films through sputtering using a sputter coater 208 HR (Cressington Scientific Instruments, Watford, UK). The approximate thickness of the sputtered electrodes was 50 nm. The dielectric permittivity was measured as a function of temperature in a frequency range 10^3^–10^6^ Hz using a Solartron 1260 impedance analyzer with a dielectric interface of 1296 (Solartron Analytical, Farnborough, UK). The measurements were performed upon heating, as well as upon cooling within a temperature range of 270 K to 370 K, and the data points were collected every 2 K. The polarization hysteresis curves were measured using a TF Analyzer 2000 (aixACCT, Aachen, Germany) at temperatures between 298 and 323 K. The applied voltage was a triangular wave function with a frequency of 10 Hz. The thermophysical properties were investigated through differential scanning calorimetry measurements that were performed using a DSC-204 (Netzsch, Selb, Germany) setup.

## 3. Results

### 3.1. Thermal Properties

The impact of compositional variation on the thermal properties (melting and recrystallization temperatures) of the polymer powder was studied. Aluminum crucibles were used and 10 mg of polymer powder was encapsulated in it. The curves were measured with a heating/cooling rate of 10 K min^−1^. The melting and recrystallization temperatures of all samples were determined from the positions of the endothermic and exothermic peaks on the thermograms (Figure S2), and are enlisted in Table 2. The melting temperature of the studied compositions varies from 394 K to 405 K, while the recrystallization temperature lies between 368 K and 382 K.

### 3.2. Relaxor Properties

**Figure 2:**
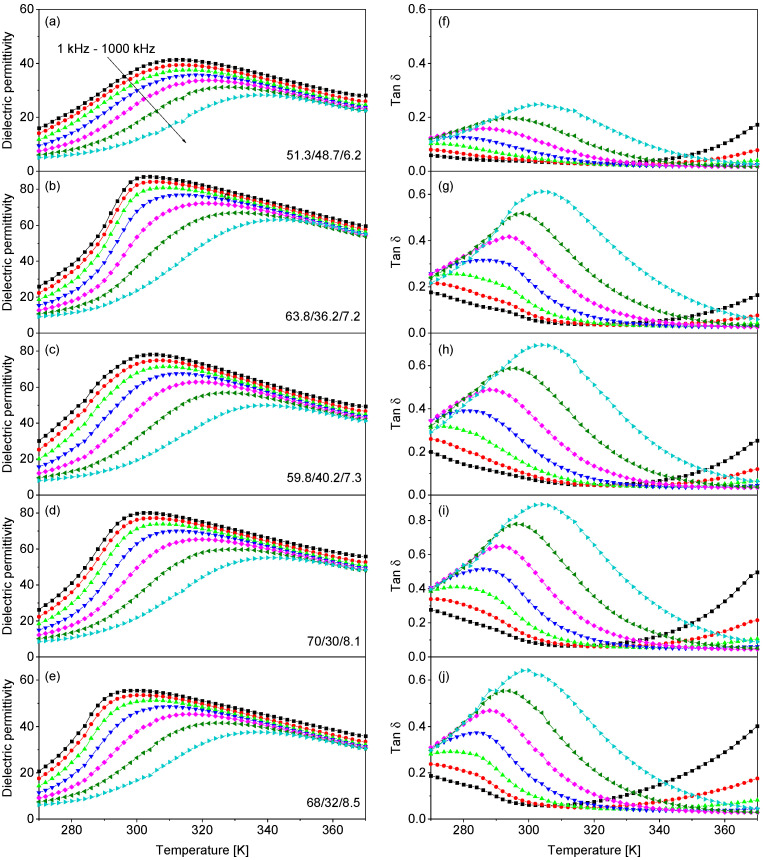
Temperature dependence of the dielectric permittivity (**a**–**e**) and dielectric loss tangent (**f**–**j**) of P(VDF_x_-TrFE_1-x_-CFE_y_) films measured at frequencies from 1 kHz to 1 MHz upon cooling.

Figure 2 shows the temperature dependence of the relative dielectric permittivity and dielectric loss tangent of the samples under study, measured at frequencies from 1 kHz to 1 MHz upon cooling. All samples manifest broad peaks in the dielectric permittivity and loss tangent. With increasing frequency, the positions of these peaks shift towards higher temperatures, the values of the dielectric permittivity become smaller, while the dielectric loss tangent increases. Such a behavior is a distinctive feature of relaxor materials [9]. For the same frequency, the dielectric permittivity peak occurs at a lower temperature for the composition with a higher CFE content. The maximal relative dielectric permittivity lies in the range of 45 to 90, which is in good agreement with the previous reports [22].

The permittivity-temperature curves were further analyzed to characterize the relaxor behavior in detail. In order to estimate the degree of the relaxor behavior, the modified Curie-Weiss law is often used to approximate the temperature dependence of *ε(T)* above *T_m_* (Equation (3)) [23].

(3) $$\frac{\varepsilon_{m}}{\varepsilon \left(T\right)}-1={\left(\frac{T-T_{m}}{2\sigma }\right)}^{\gamma }$$

**Figure 3:**
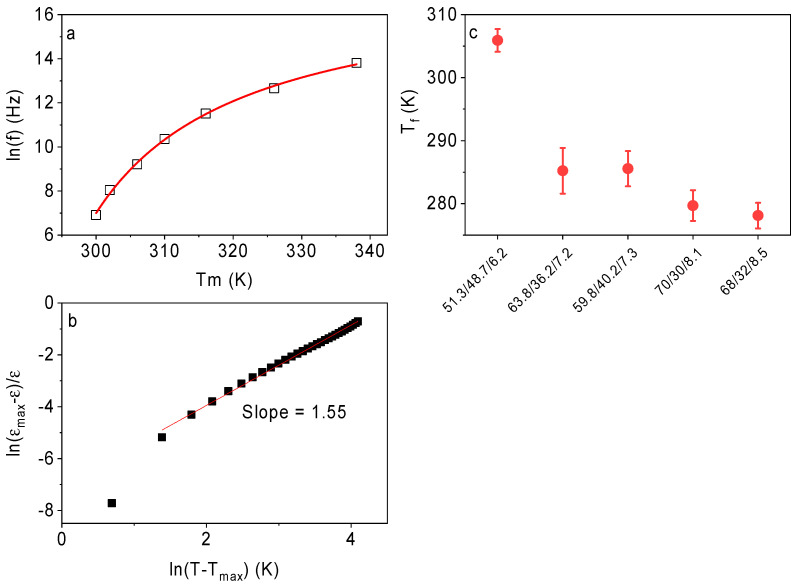
Examples of (**a**) the Vogel-Fulcher and (**b**) the modified Curie-Weiss law fitting for the P(VDF-TrFE-CFE) 68/32/8.5 film. (**c**) The freezing temperatures, *T_f_*, of the studied samples.

Here, *ε_m_* is the maximal value of the dielectric permittivity, *T_m_* is the temperature at the maximum permittivity, the parameter *σ* describes the broadening of the dielectric peak, and the exponent *γ* reflects the degree of the relaxor behavior. For classical ferroelectric materials, the value of *γ* is equal to 1; while for the canonical relaxors, its value approaches 2 [24]. An example of the fit of *ε(T)* by Equation (3) for the P(VDF-TrFE-CFE) 68/32/8.5 film is shown in Figure 3b. The fitting curve matches the experimental data well. The best fit values for *γ* for the studied polymers lie between 1.5 and 1.7 (Table 3), which confirms the strong relaxor behavior.

Unlike ferroelectric materials, the relaxors do not undergo a phase transition into the ferroelectric state at the Curie temperature. Instead, the slowing-down of the PNRs dynamics results in a transition into a glassy-like state with short-range correlated polarization (non-ergodic relaxor state) at the freezing temperature, *T_f_* [9]. The freezing temperature can be determined by using the Vogel-Fulcher equation (Equation (4)) [25].

(4) $$f=f_{0}\times exp\left(\frac{E_{a}}{k\left(T_{m}\left(f\right)-T_{f}\right)}\right)$$

Here, *f* is the frequency of the applied electric field, *T_m_(f)* is the corresponding temperature of the maximum of the dielectric permittivity, *T_f_* is the freezing temperature, *E_a_* is the activation energy, *f*_0_ is the attempt frequency, and k is the Boltzmann constant. Figure 3a shows an example of the Vogel-Fulcher fit of *T_m_(f)* for the P(VDF-TrFE-CFE) 68/32/8.5 film. The best-fitting parameters for the studied samples are given in Table 3. Figure 3c illustrates the variation in the freezing temperature for the different compositions. One can see that with an increase in the CFE content, the freezing temperature decreases, while the activation energy increases. In general, it can be concluded that the increase in the CFE content expands the range of the ergodic relaxor state towards lower temperatures.

### 3.3. Polarization Hysteresis Behavior

**Figure 4:**
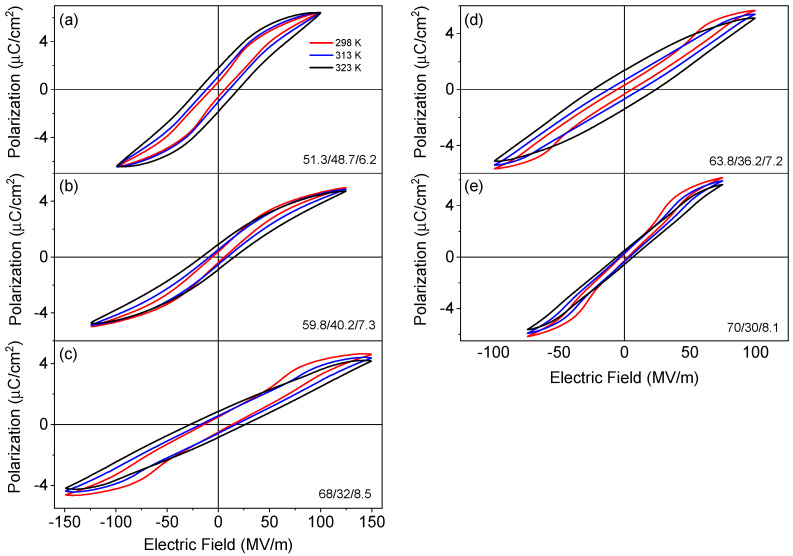
Polarization hysteresis loops of the P(VDF-TrFE-CFE) 51.3/48.7/6.2 (**a**), 59.8/40.2/7.3 (**b**), 68/32/8.5 (**c**), 63.8/36.2/7.2 (**d**), and 70/30/8.1 (**e**) films measured as a function of temperature at 10 Hz (triangular wave) on cooling.

The polarization hysteresis loops were measured upon heating as well as cooling. The results obtained during the cooling cycle are shown in Figure 4. The 59.8/40.2/7.3 terpolymer exhibits slim polarization hysteresis loops typical for relaxors (Figure 4b). A particular feature of other compositions is a jump of the polarization above a certain electric field value, which results in the DHL shape (Figure 4). Such DHL-shape was already reported for some P(VDF-TrFE-CFE) polymers, e.g., for 59.2/33.6/7.2 (63.8/36.2/7.8 in our notation). The DHL was explained in terms of a reversible, electric field-induced relaxor to ferroelectric phase transition [12].

**Figure 5:**
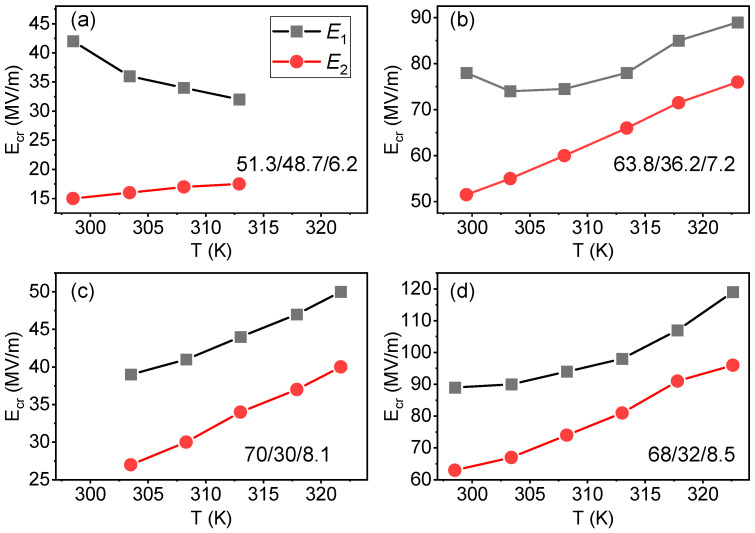
Temperature dependences of the critical fields, *E*_1_ and *E*_2_, corresponding to the field-induced transitions between relaxor and ferroelectric states for the P(VDF-TrFE-CFE) 51.3/48.7/6.2 (**a**), 63.8/36.2/7.2 (**b**), 70/30/8.1 (**c**), and 68/32/8.5 (**d**) polymer films.

We have taken a detailed look at the polarization curves shown in Figure 4 to evaluate the critical fields corresponding to the transition to the ferroelectric state, *E*_1_, and back to the relaxor state, *E*_2_. These fields were estimated from the maxima of the field derivative of polarization. Figure 5 shows the temperature dependences of *E*_1_ and *E*_2_ for the studied compositions. It can be observed that the P(VDF-TrFE-CFE) 63.8/36.2/7.2 (Figure 5b) and P(VDF-TrFE-CFE) 68/32/8.5 (Figure 5d) samples have the largest critical field values, (*E*_1_ = 80–90 MV·m^−1^) at room temperature; while for the P(VDF-TrFE-CFE) 51.3/48.7/6.2 (Figure 5a) the transition to the ferroelectric state occurs already at 40 MV·m^−1^. For all the studied polymers, both *E*_1_ and *E*_2_ increase with temperature except for P(VDF-TrFE-CFE) 51.3/48.7/6.2, where *E*_1_ goes slightly down upon heating.

When we refer to the field-induced relaxor–ferroelectric transition in such inorganic relaxors as (Pb,La)(Zr,Ti)O_3_ [26] or Na_0.5_Bi_0.5_TiO_3_-BaTiO_3_, [27] it can be seen that the critical field value decreases with temperature in the non-ergodic relaxor state, but increases with temperature in the ergodic relaxor state. The non-ergodic relaxor state is the state below the freezing temperature, where the interaction between PNRs blocks their reorientation and related dynamics. According to the estimations from the dielectric data, the freezing temperature of the P(VDF-TrFE-CFE) 51.3/48.7/6.2 sample lies at 306 K, i.e., the range where DHL is observed is around the transition from the non-ergodic to the ergodic relaxor state. On the other hand, other compositions are in the ergodic relaxor state far from the freezing temperature and we observe a growing critical field within a temperature range of 298 K to 313 K, while above 313 K, a single hysteresis loop (SHL) is observed.

Another remarkable feature of the polarization hysteresis loops in all studied samples is the broadening of the hysteresis loops with temperature, which leads to an increase in the measured values of the remnant polarization as well as the coercive field. This broadening is related to the contribution of leakage current [28], which increases with temperature, as can also be seen in the dielectric loss curves at low-frequency (1 kHz) in Figure 2. In the case of polymers, this leakage current can be related to the field-induced transport of electronic and ionic charges. In some publications, this increase in the extrinsic polarization was considered as a signature of a negative electrocaloric effect (ECE) [29,30,31].

The ECE, which is the change in the temperature/entropy of a dielectric material under an adiabatically/isothermally applied electric field, has gained popularity as an environmentally friendly technology for the development of compact solid-state refrigeration and air-conditioning systems [32,33,34,35]. Since direct electrocaloric measurements need particular devices and modifications, the indirect method is more widely used [32,36,37]. The indirect method is based on the Maxwell relation *(∂S/∂E)_T_ = (∂P/∂T)_E_*. In this case, the isofield temperature dependences of the polarization obtained from the hysteresis loops are used to evaluate the electrocaloric change of the temperature

(5) $$\Delta T_{EC}=-\int_{E_{1}}^{E_{2}}\left(T/C_{E}\right){\left(\partial P/\partial T\right)}_{E}dE$$

From this equation, it is obvious that a positive temperature derivative of polarization implies a negative electrocaloric effect, as was reported by some groups [29,30,31]. While the direct electrocaloric measurements of similar compositions only show a positive electrocaloric effect [38,39]. Thus, the leakage-related extrinsic contribution to the polarization may lead to erroneous evaluation of the electrocaloric effect in ferroelectric and relaxor polymers [40].

As we mentioned already, the relaxor behavior in P(VDF-TrFE-CFE) is promoted by the incorporation of bulky CFE monomers, which results in an increasing distance between the P(VDF-TrFE-CFE) chains. This weakens the cooperative polarization of the P(VDF-TrFE) dipoles and reduces the size of the ferroelectric domains to the nanoscale. The structure of relaxor P(VDF-TrFE-CFE) contains more or less random TmG (m ≤ 4) sequences, and the FE structure contains relatively long Tn (*n* > 4) sequences (T and G represent the trans and gauche conformations, respectively) [5].

The CFE monomers possess their own dipole moment and can serve as dipole defect-pinning centers, similar to dipole defects built by oxygen vacancies and acceptor impurities in perovskite ferroelectrics [41]. When the magnitude of the applied electric field is large enough, the dipoles of CFE may rotate, promoting the switching of the surrounding dipoles and the formation of a ferroelectric state with large domains [5]. As the field is reduced and eventually removed, these FE domains transform back to the relaxor phase. In the ergodic relaxor state, the PNRs are relatively free to rotate and can be easily aligned. Meanwhile, the thermal agitation breaks this alignment and therefore the critical field, E_1_, increases upon heating. When the sample is in the non-ergodic relaxor state, the frozen PNRs are relatively difficult to be reoriented, but they become less blocked on approaching the freezing temperature; therefore, we observe that the critical field decreases upon heating for P(VDF-TrFE-CFE) 51.3/48.7/6.2. The disappearance of the DHL with temperature can be attributed to the fact that the polymer chains become mobile with temperature and the pinning effect due to the presence of CFE in the polymer chain becomes less pronounced. However, this process is also obscured by the broadening of the hysteresis loops due to the increased conductivity.

### 3.4. Energy Storage Properties

The stored and recoverable electrical energy densities are given by Equations (1) and (2). The stored energy cannot be fully recovered due to the leakage current and the hysteresis losses (*U_loss_*). The energy storage efficiency, *η*, is defined as the ratio between the discharged and stored energy of the capacitor (Equation (6)) [42,43].

(6) $$\eta =U_{discharged}/U_{stored}=U_{discharged}/\left(U_{discharged}+U_{loss}\right)\;$$

All these characteristics can be deduced from the unipolar polarization hysteresis curves recorded for a different maximum electric field (Figure S3).

**Figure 6:**
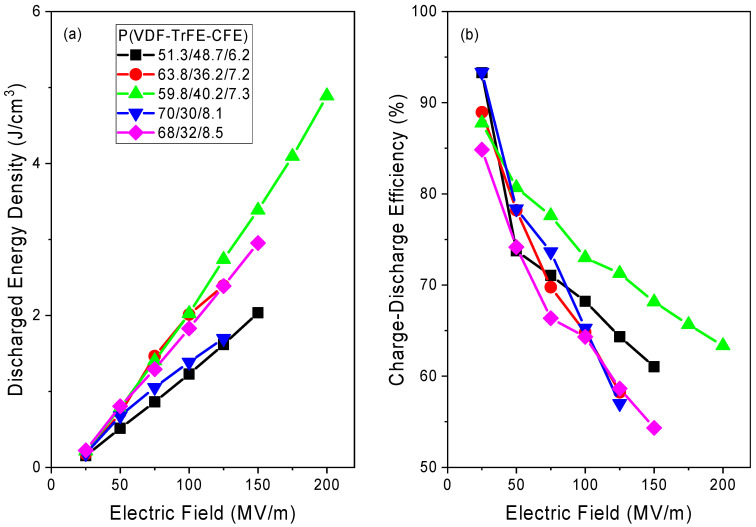
The discharged energy densities (**a**) and charge–discharge efficiencies (**b**) plotted against maximum applied electric field (estimated from the unipolar hysteresis loops shown in Figure S3).

Figure 6 shows the maximum field dependences of the discharged energy density and energy storage efficiency calculated at room temperature. All compositions show a linear increase in the discharged energy density with the applied electric field. However, their energy storage efficiency decreases with the increasing field. There is a tradeoff between the discharged energy density and the energy storage efficiency due to the hysteresis losses. As is clear from Figure 6, the P(VDF-TrFE-CFE) = 59.8/40.2/7.3 sample outperforms the other compositions in terms of the discharged energy density as well as the energy storage efficiency. It shows the highest energy density that reaches *η* = 5 J·cm^−3^ at a field of 200 MV·m^−1^, while retaining an efficiency of around 63%. The P(VDF-TrFE-CFE) 63.8/36.2/7.2 and 68/32/8.5 films show the comparable discharged energy density at a field of 100 MV·m^−1^, but have a lower energy storage efficiency due to the DHL behavior. In Table 4, the discharged energy density of different compositions at a field of 100 MV·m^−1^ are compared with data reported in the literature. One can see that the discharged energy density of the polymers studied in this work is consistent with the previously reported values.

## 4. Conclusions

Dielectric and polarization properties of P(VDF-TrFE-CFE) polymers with different compositions are compared. All samples show a similar degree of relaxor behavior. Increasing the CFE content shifts the freezing temperature down and stabilizes the ergodic relaxor state at room temperature. The application of a strong enough electric field induces a transition into a ferroelectric state, as manifested in double hysteresis loops. The value of the critical electric field depends on the proximity to the freezing temperature. Correct indirect measurements of the electrocaloric effect are impossible in the studied materials due to the charge leakage extrinsic contribution to polarization enhanced at higher temperatures. Direct electrocaloric measurements are necessary. In particular, it will be interesting to study a relation between the field-induced transition to the ferroelectric state and the electrocaloric effect at the corresponding field values.

The characteristics of the polymer films for energy storage application were tested. A maximum energy density of 5 J·cm^−3^ at 63% charge–discharge efficiency and a field of 200 MV·m^−1^ is observed for the new composition P(VDF-TrFE-CFE) 59.8/40.2/7.3. To further improve these characteristics, composites with inorganic fillers can be developed based on these terpolymers. Such studies are now in progress.

## Figures and Tables

**Table 1:** The molar content of each component in the studied P(VDFx-TrFE_1-x_-CFE_y_) compositions. It is important to note that the molar contents are mentioned in a way that the amount of VDF and TrFE adds up to 100.

VDF (mol. %)	TrFE (mol. %)	CFE (mol. %)
51.3	48.7	6.2
63.8	36.2	7.2
59.8	40.2	7.3
70	30	8.1
68	32	8.5

**Table 2:** The melting and recrystallization temperatures of the studied compositions estimated from DSC measurements.

P(VDF_x_-TrFE_1-x_-CFE_y_) (mol. %)	Melting Temperature (K)	Recrystallization Temperature (K)
51.3/48.7/6.2	404	382
63.8/36.2/7.2	394	368
59.8/40.2/7.3	405	379
70/30/8.1	399	370
68/32/8.5	398	372

**Table 3:** Parameters describing the relaxor behavior of the studied the P(VDF_x_-TrFE_1-x_-CFE_y_) films.

P(VDF_x_-TrFE_1-x_-CFE_y_) (mol. %)	Degree of Relaxor Behavior, *γ*	Freezing Temperature, *T_f_* (K)	Fitting Parameters for the Vogel-Fulcher Equation	
			ln(*f*_0_)	*E*_a_ (meV)
51.3/48.7/6.2	1.61	306 ± 2	15.8 ± 0.9	6 ± 2
63.8/36.2/7.2	1.52	285 ± 4	16.3 ± 0.8	14 ± 4
59.8/40.2/7.3	1.64	286 ± 3	17.3 ± 0.7	16 ± 4
70/30/8.1	1.50	280 ± 2	17.5 ± 0.5	21 ± 3
68/32/8.5	1.55	278 ± 2	17.6 ± 0.5	20 ± 3

**Table 4:** Comparison of the discharged energy density for different P(VDF-TrFE-CFE) compositions.

P(VDF_x_-TrFE_1-x_-CFE_y_)	Electric Field (MV·m^−1^)	Discharged Energy Density (*U_discharged_*) (J·cm^−3^)	Reference
51.3/48.6/6.2	100	1.2	This work
63.8/36.2/7.2	100	2	This work
64/36/7.2	100	1.8	[44]
70/30/8.1	100	1.38	This work
70/30/8.1	100	2	[44]
63/37/8.1	100	1.7	[45]
68/32/8.5	100	2	This work
59.8/40.2/7.3	100	2	This work

## Data Availability

The data presented in this study are available on request from the corresponding author.

## Supplementary Materials

The following are available online at https://www.mdpi.com/article/10.3390/polym13081343/s1, Figure S1: The ternary compositional diagram of P(VDF-TrFE-CFE) indicating the studied compositions, Figure S2: Heat flow curves measured by DSC upon heating (above) and cooling (below), where the peaks correspond to melting and recrystallization, respectively, Figure S3: Unipolar polarization hysteresis curves measured as a function of voltage at 10 Hz and room temperature.
