# Supplementary material for: Effect of Composition on Polarization Hysteresis and Energy Storage Ability of P(VDF-TrFE-CFE) Relaxor Terpolymers
Source: Polymers (Basel). 2021 Apr 20;13(8):1343. doi: 10.3390/polym13081343 (PMC8073565; doi:10.3390/polym13081343)
Supplement: Supplementary file 1 [file polymers-13-01343-s001.zip › polymers-1174520-supplementary.pdf]

polymers

MDPI

Supplementary Materials

Effect of composition on polarization hysteresis and energy storage ability of P(VDF-TrFE-CFE) relaxor terpolymers

Yusra Hambal[1], *Vladimir V. Shvartsman*[1*], *Daniil Lewin*[1], *Chieng Huo Huat*[1], *Xin Chen*[2], *Ivo Michiels*[1], *Qiming Zhang*[2], *Doru C. Lupascu*[1]

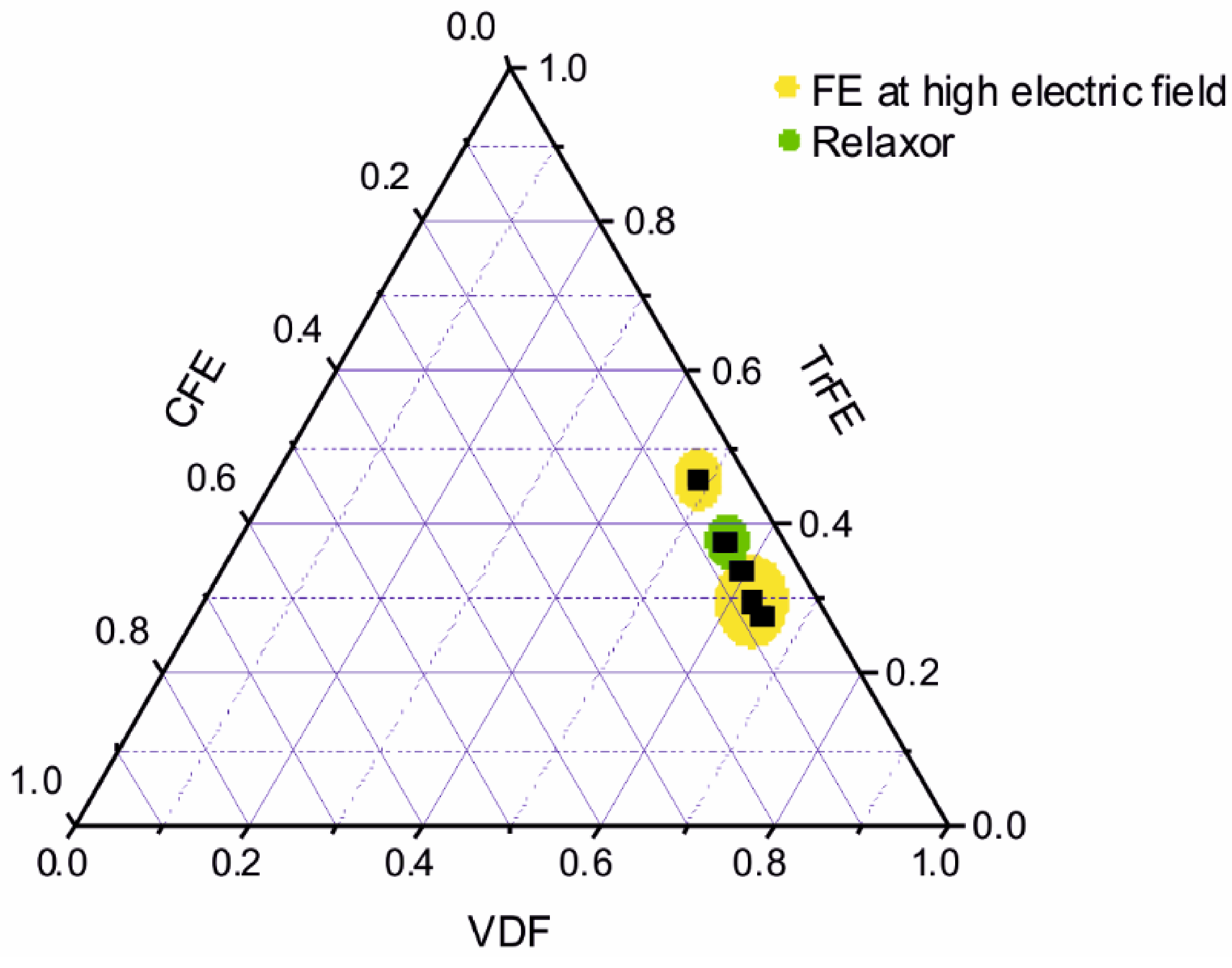

Figure S1. The ternary compositional diagram of P(VDF-TrFE-CFE) indicating the studied compositions.

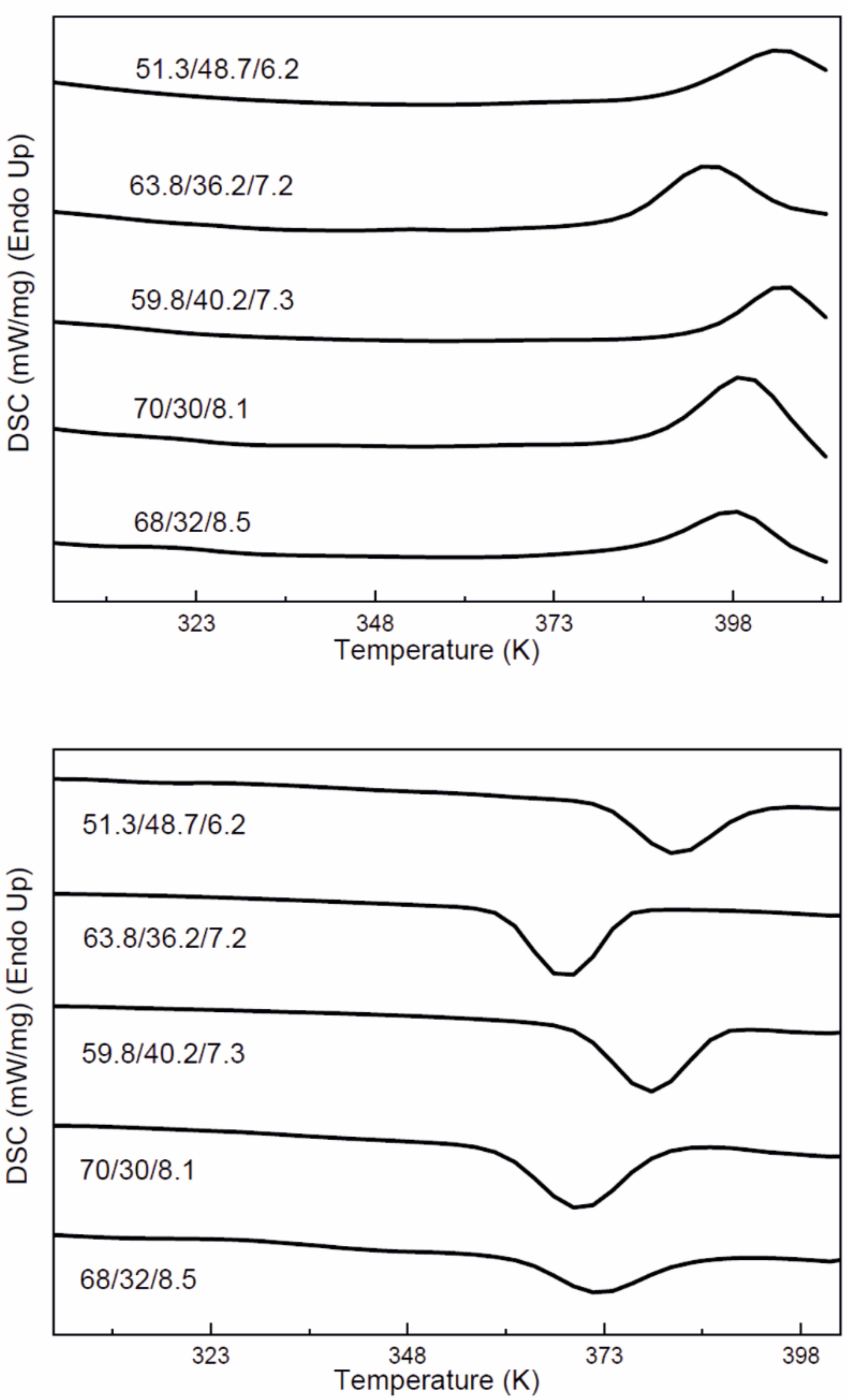

Figure S2. Heat flow curves of the P(VDF$_x$-TrFE$_{1-x}$-CFE$_y$) polymer films measured by DSC upon heating (above) and cooling (below), where the peaks correspond to melting and recrystallization, respectively.

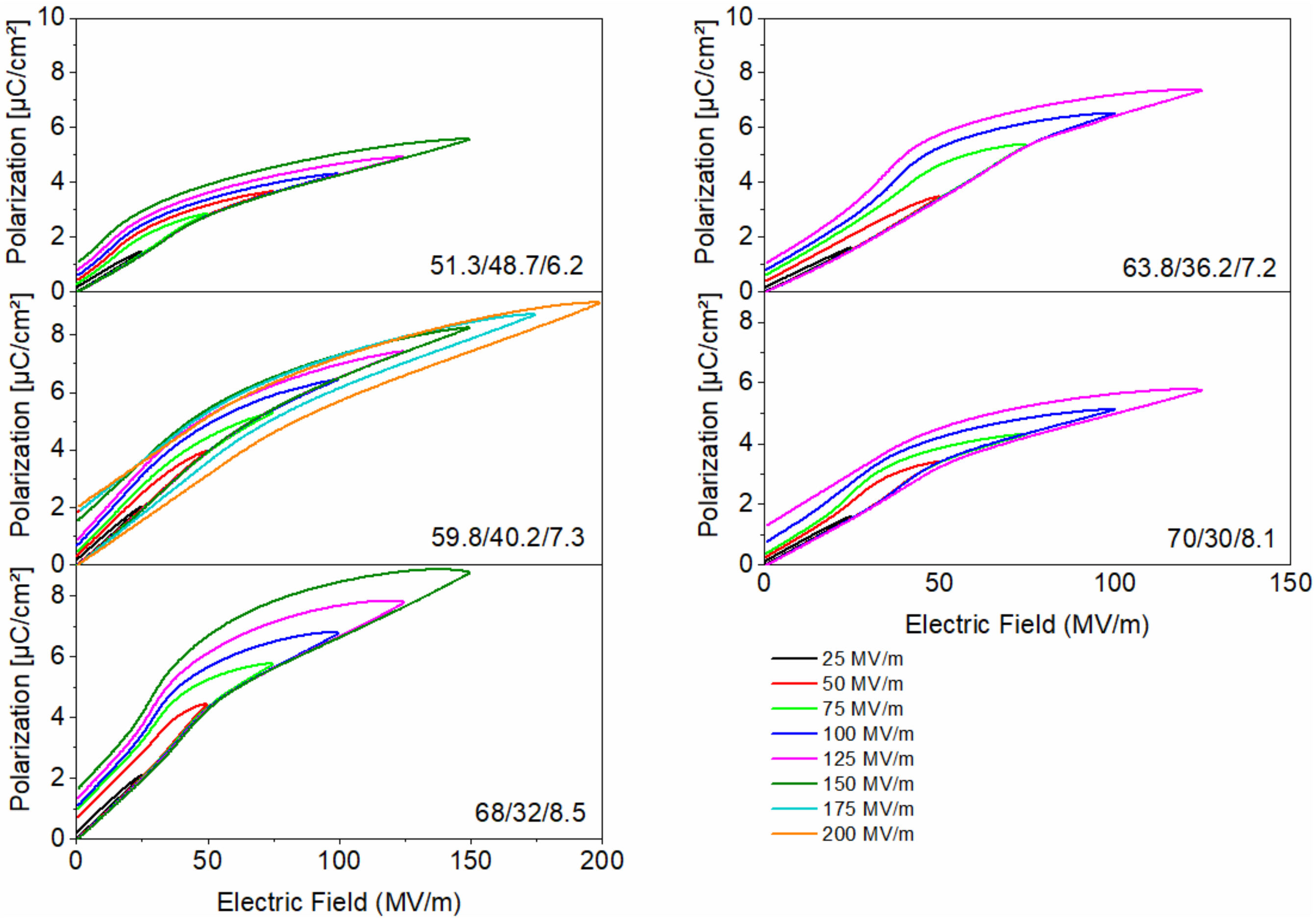

Figure S3. Unipolar polarization hysteresis curves of the P(VDF_x-$TrFE_{1-x}$-CFE_y) polymer films measured as a function of voltage at 10 Hz and room temperature.
